# Efficiency at Olympia

**Published:** 1921-02-19

**Authors:** 


### EFFICIENCY AT OLYMPIA.

### BY A SOCIAL WORKER.

To be an educated person, Herbert Spencer long ago taught us, means to be able to do what you ought, when you ought, and as you ought, whether you want to do it or not. This, we take it, is the ideal which EFFICIENCY imprisons in its ten letters.

At Olympia it is worked out in a delightfully logical way. To begin with, journalists, invited to the private view, had a practical exemplification if they went on succeeding days. A chaos of dust, tarpaulins, and unattached wheels had, like the mixed feathers of the fairy tale, sorted themselves at the top of Fairy Efficient's wand, and in the rows of compact platforms each picture now tells its story. If you are a woman you learn, step by step, how to order a home efficiently and to have time for other efficient labour (you can even have a home; an inviting notice announces that at our newest Garden City there are houses to be let and sold). You will burn smokeless coal in it, abolish knife machines by favour of stainless steel, fit your kitchen with labour-saving appliances and your tea-table with clean milk, and at the excellent bookstall of the National Council of Women buy manuals of instruction in every kind of domestic craft, and first-rate volumes on sociology to enable you to become also an efficient citizen.

If you are a man you can learn how to fit up your study or office with contrivances so labour-saving that surely they must add to unemployment by reducing the need for clerks and other staff. When a machine will add and multiply and calculate interest for you, when the appliances for indexing are so delightful that the fingers itch to index something then and there, when furniture touches the ideal of comfort and convenience, and the dictaphone suggests that you can enjoy it undisturbed, there seems only one more need for the business man, the ideal employee. Olympia bristles with offers of trainings which are thoroughly up to date: we note that employers and foremen study psychology and workers are encouraged to concentrate. Finally, for an extra shilling you can see how Royalty and some great Efficients of to-day design rooms for work, and that shilling will help the Middlesex Hospital to isolate and destroy bacteria that tend to inefficiency.

So far the fun of the fair, but step into the Annexe and face our great national problem, the restoration to efficiency of the war's disabled victims. You pass, before you enter this section, the stall where Sir A. Pearson appeals for more Sunshine Homes for the blinded babies.

There are minor problems, too. Why do not our spinsters spin? A hand-loom worker, surrounded by piles of the really beautiful soft tweeds which cost only 10s. 6d. the yard, says regretfully that his weft is machine-spun. A Sussex man will make sound new wool-wheels for £2 each; a girl or woman can do part-time work at home, in the garden if she will. Girls will knit now—that is new. Who will start a fashionable craze for the wheel? One appeal there is to the voluntary worker. The Kensington war supply for surgical appliances in papier maché is now a peace supply for wonderful contrivances in celluloid, light in the extreme, yet strong. This craft and other work done there is fascinating and of great value. Who will help?

